# Supplementary material for: The association between shame and substance use in young people: a systematic review
Source: PeerJ. 2015 Jan 22;3:e737. doi: 10.7717/peerj.737 (PMC4312064; doi:10.7717/peerj.737)
Supplement: Supplemental Information 1 [file peerj-03-737-s001.doc]

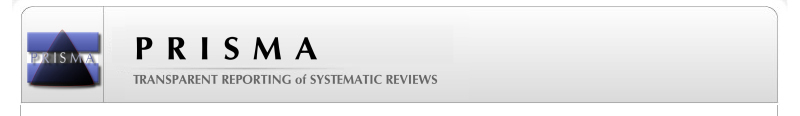
**PRISMA 2009 Flow Diagram**

**Screening**

**Included**

**Eligibility**

**Identification**

Records identified through database searching
(n = 735)

Additional records identified through other sources
(n = 0)

Records after duplicates removed
(n = 220)

Records screened
(n = 220)

Records excluded
(n = 131)

Full-text articles assessed for eligibility
(n = 89)

Full-text articles excluded, with reasons
(n = 83)

Studies included in qualitative synthesis
(n = 6)
